# A variant reference data set for the Africanized honeybee, *Apis mellifera*

**DOI:** 10.1038/sdata.2016.97

**Published:** 2016-11-08

**Authors:** Samir M. Kadri, Brock A. Harpur, Ricardo O. Orsi, Amro Zayed

**Affiliations:** 1 Departamento de Produção Animal, Faculdade de Medicina Veterinária e Zootecnia, Univ Estadual Paulista, UNESP, Botucatu, São Paulo 18618681, Brasil; 2 Department of Biology, Faculty of Sciences, York University, Toronto, Ontario M3J1P3, Canada

**Keywords:** Agricultural genetics, Comparative genomics, Entomology, Genetic variation, DNA sequencing

## Abstract

The Africanized honeybee (AHB) is a population of *Apis mellifera* found in the Americas. AHBs originated in 1956 in Rio Clara, Brazil where imported African *A. m. scutellata* escaped and hybridized with local populations of European *A. mellifera*. Africanized populations can now be found from Northern Argentina to the Southern United States. AHBs—often referred to as ‘Killer Bees’— are a major concern to the beekeeping industry as well as a model for the evolutionary genetics of colony defence. We performed high coverage pooled-resequencing of 360 diploid workers from 30 Brazilian AHB colonies using Illumina Hi-Seq (150 bp PE). This yielded a high density SNP data set with an average read depth at each site of 20.25 reads. With 3,606,720 SNPs and 155,336 SNPs within 11,365 genes, this data set is the largest genomic resource available for AHBs and will enable high-resolution studies of the population dynamics, evolution, and genetics of this successful biological invader, in addition to facilitating the development of SNP-based tools for identifying AHBs.

## Background & Summary

The European honeybee (*Apis mellifera*) was introduced to North and South America from Old World populations in the early 18th century^1^. In its native range, the honeybee is divided geographically and genetically into five ancestral lineages—the M and C lineages of Europe, the A lineage of Africa, and the Y and O lineages of Asia^2–7^—that encompass approximately 22 subspecies^7^.

European settlers, in the early 18th century, introduced subspecies of the M lineage (*A. m. mellifera* and *A. m. iberica*) into North America^8,9^. By the 20th century, C lineage (*A. m. ligustica and A. m. carnica*) and some O lineage (*A. m. caucasia*) subspecies were introduced^8,9^. It was during this century that Brazilian beekeepers first imported honeybees, chiefly *A. m. mellifera* and *A. m. carnica*, followed by *A. m. ligustica* and *A. m. caucasia*^10^. These subspecies were used exclusively in Brazil until 1956 when *A. m. scutellata* was imported from Africa for genetic research. Mated *A. m. scutellata* queens arrived from South Africa^11^ to breeding stations in Rio Claro, São Paulo, Brazil. The intention of the breeding program was to crossbreed *A. m. scutellata* with commercial stock to serve as a base population in selection programs^12^. Famously, queens and drones escaped and hybridized with the existing population^12,13^. These hybrids became one of the most astounding insect invaders in recent history; feral populations of the ‘Africanized’ bees retained the highly defensive trait of their African ancestors and are now the most common honeybee found from Central South America (Brazil and Northern Argentina) to Mexico and the southern United States^14^.

The sequencing of the honeybee genome in 2006 (ref. 15) was a landmark for the field of sociogenomics and has created valuable resources for the beekeeping industry^16–19^, but because this genome was derived from a typical admixed North American honeybee^15^ it provides little information about the underlying genetic variation present in Africanized populations.

Here, we present the genomes of 360 AHBs from Brazil along with a reference SNP database for this population. Genomic resources for AHBs will be beneficial for both pure and applied research questions. First, there is a growing need to quickly and reliably detect Africanized colonies to secure international trade in honeybees^16,19^. Second, Africanized bees are highly defensive and they are commonly used for studying the genetics of nest defence^20^. Finally, Africanized bees are highly invasive: within the last 60 years they have become the most common genotype across much of the Southern United States^14^. There is evidence that adaptive introgression occurred during their invasion^21^.

## Methods

### Sampling and sample information

We collected 12 diploid worker bees from the brood frames of each of 30 Africanized honeybee colonies from four apiaries all located at Iaras city, São Paulo, Brazil (Table 1), located approximately 200 km from Rio Claro. These colonies were obtained from natural swarms within the State of São Paulo where the Africanized honeybee invasion began. We performed a single DNA extraction for each colony by pooling ¼ of a thorax from each of the 12 workers^22^. We used the Mag-Bind Blood DNA kit (Omega Biotek Store) with the manufacturer's recommended protocol to extract an average of 4.71±1.42 μg of high-quality DNA from each of the colonies.

DNA samples (one pooled sample of 12 workers from each colony) were submitted to The Centre for Applied Genomics (Toronto, ON) for library preparation and high-throughput sequencing. In brief, DNA was quantified by Qubit HS assay and 200 ng of DNA was used as input material for the TruSeq Nano DNA Sample Preparation protocol (Illumina, Inc.) following Illumina's recommendation. DNA was sheared to 550-bp on average using a Covaris S2 system (Duty cycle: 10%; Intensity 2; Burst per second: 200; Treatment time: 44 s; Mode: Frequency sweeping). The sheared DNA was end-repaired and the 3' ends were adenylated prior to ligation of the TruSeq adapters. The library was enriched by PCR using different indexed adapters to allow for multiplex sequencing using the following conditions: 95 °C for 3 min followed by 8 cycles of 98 °C for 20 s, 60 °C for 15 s and 72 °C for 30 s, and finally an extension step at 72 °C for 5 min.

Final TruSeq Nano DNA genomic libraries were validated on a Bioanalyzer 2100 DNA High Sensitivity chip (Agilent Technologies) for size and by qPCR using the Kapa Library Quantification Illumina/ABI Prism Kit protocol (KAPA Biosystems) for quantities. Ten libraries were pooled in equimolar quantities and sequenced on a HiSeq 2500 platform on a high throughput flowcell with the Illumina TruSeq V4 sequencing chemistry following Illumina’s recommended protocol to generate paired-end reads of 150-bases in length.

### Genome alignment and variant calling

**Figure 1:**
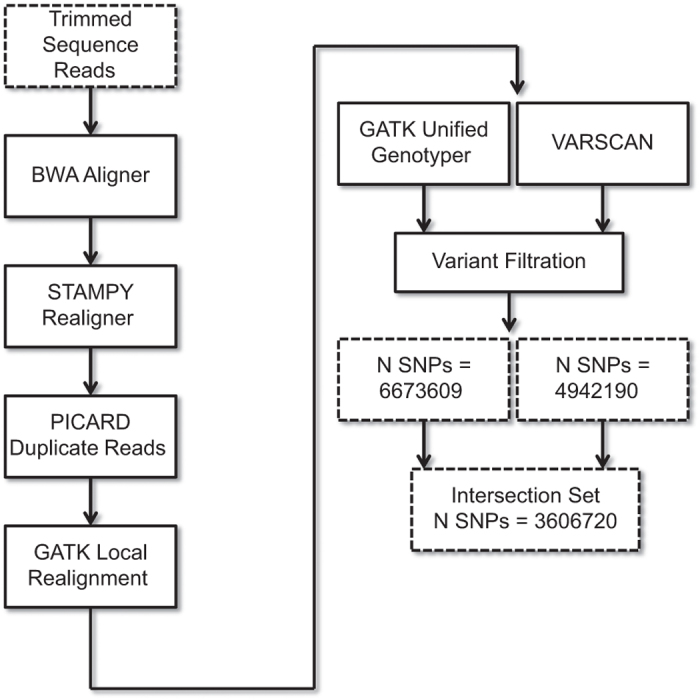
Overview of alignment and SNP calling pipeline.

Each colony’s sequenced reads (*N*=30 colonies) were trimmed of Illumina Adaptors using Trimmomatic v0.32 then aligned to the most recent version of the honeybee genome (Amel_4.5 (ref. 23)) using BWA aligner v0.7.5 (ref. 24). Paired alignments were then merged with SAMTOOLS v 0.1.19 (ref. 25) and re-aligned using STAMPY v1.0.21 (ref. 26) with divergence (--d) set at 0.02. We marked and removed duplicate reads with PICARD v 1.141 and re-aligned around indels using GATK IndelRealigner v 3.1 (ref. 27) (Fig. 1; Data Citation 1).

To identify all variants found within our samples, we used two independent variant callers (Fig. 1): VARSCAN^28^ v2.3.7 and GATK UnifiedGenotyper (Data Citations 2 and 3) We used GATK with --ploidy 2 to identify only the location of variants and were unconcerned with specific genotype calls. We called all variant sites in GATK and VARSCAN using default parameters. We removed indels and all SNPs within 10 bp of indels, removed all unmapped scaffolds (Scaffolds 17.XXX or GroupUn) and mitochondrial sequence (Scaffold 18.1), and removed SNPs of low quality (Q<25) or in areas of low genomic complexity, thus reducing the potential for calling erroneous SNPs due to paralogous sequence or misaligned reads^3^ (Fig. 1). We retained all SNPs that were identified using both variant callers and that passed our conservative filtering procedures, above. Because our data consist of pooled sequence for 30 colonies, we report the allele frequency at each site as called by VARSCAN in Variant Call Format (Data Citation 3).

### SNP validation: Population differentiation and admixture

To quantify differentiation among contemporary Africanized populations in Brazil and ancestral honeybee populations, we used POPOOLATION2 v1.201 (ref. 29). We created a single input file containing our Africanized bee samples pooled into a single alignment as well as population-pooled alignments from ancestral honeybee populations from Africa (A lineage, *N*=11) and Europe (M lineage, *N*=9; C lineage *N*=9). The latter sequence data were obtained from a recent honeybee population genomics study^3^ and represents ancestral populations from which Africanized populations are derived. We generated a single MPILEUP file and extracted from it the 3,606,720 SNPs identified above in our Africanized honeybee samples. We estimated pairwise population differentiation on all sites with --min-count 6 --min-coverage 100 --max-coverage 800 --min-covered-fraction 0.8.

### Code availability

We have not used any custom code and relied on previously available, validated, software packages; however, we have left our general pipeline available for re-use at the author’s GitHub (https://github.com/harpur/afz/blob/master/AHBPipeline.sh).

## Data Records

We have curated a set of 3,606,720 SNPs identified in 360 Africanized honeybees across 30 colonies (Data Citations 2 and 3). The data consist of SNPs called across the most recent honeybee reference genome (Amel_4.5 (ref. 23)) in Variant Call File format on placed scaffolds. Because we utilized a pooled-sequencing method, all variant sites include the frequency of each alternate allele call for each colony. All sequence data are also available in BAM format (Data Citation 1; Table 1) allowing subsequent researchers to use updated SNP calling and genotype software when available.

## Technical Validation

**Figure 2:**
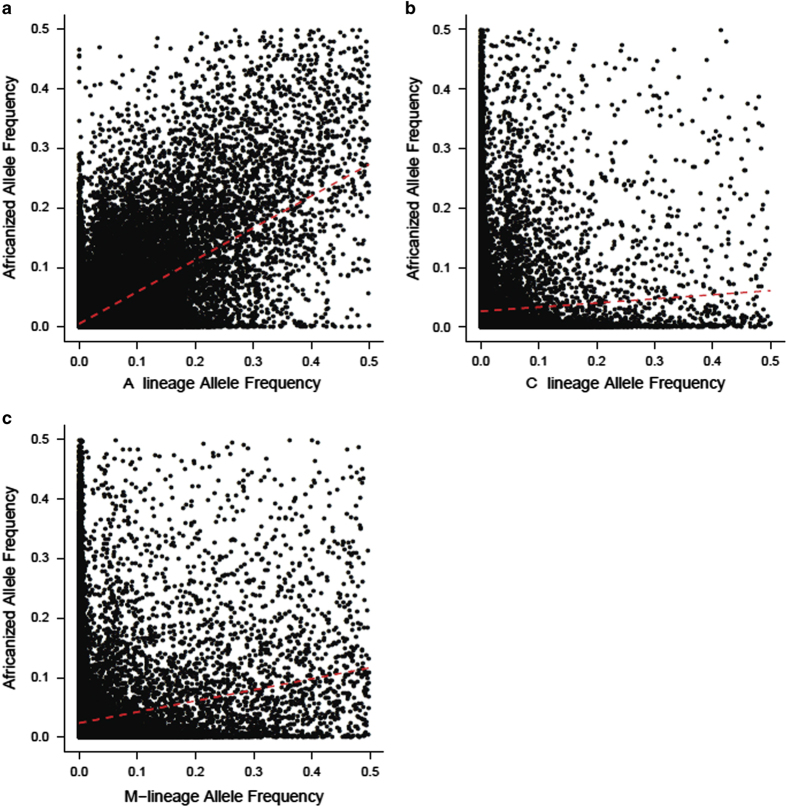
Correlation of allele frequencies between the Brazilian Africanized honeybee population and (a) A-lineage bees (b) C-lineage bees and (c) M-lineage bees. Red line shows results of linear model fit.

To validate that our samples are indeed Africanized and to confirm our SNP calls, we compared our current SNPs to those of a previous honeybee population genomics study that sequenced and analysed honeybee samples using similar methods as described herein^3^. Africanized bees are known to be derived from three of the major honeybee population groups: A, M, and C^6,16^. We found that 99.8% of the 3,606,720 SNPs found in AHBs, were also found within one or more of these ancestral populations. Africanized populations are expected to have higher A lineage ancestry relative to C and M lineage ancestry^7,16^. Using a regression model^30^, we demonstrated that allele frequencies within Africanized bees are more correlated with A lineage allele frequencies (GLM; r=0.529, *P*<2.2×10^−16^) relative to both M lineage allele frequency (r=0.102, *P*<2.2×10^−16^) and C lineage allele frequency (r=−0.08, *P*<2.2×10^−16^; Fig. 2), as we would expect from an Africanized population. As well, we find that AHB and A lineage are more similar genetically (Fst=0.02) than AHB versus M-lineage (0.04) and AHB versus C-lineage (0.05).

## Additional Information

**How to cite this article:** Kadri, S. M. *et al.* A variant reference data set for the Africanized honeybee, *Apis mellifera*. *Sci. Data* 3:160097 doi: 10.1038/sdata.2016.97 (2016).

**Publisher’s note:** Springer Nature remains neutral with regard to jurisdictional claims in published maps and institutional affiliations.

## Supplementary Material



## Figures and Tables

**Table 1:** Sample sequencing and accession information.

**Sample ID**	**Accession No.**	**Average sequencing depth**	**Location**
HDB139	SAMN05194651	29.00	22° 53′ 56′′ S; 49° 10′ 24′′ W
HDB179	SAMN05194655	29.01	22° 52′ 02′′ S; 49° 09′ 07′′ W
HDB199	SAMN05194661	11.67	22° 53′ 56′′ S; 49° 10′ 24′′ W
HDB303	SAMN05194664	12.53	22° 50′ 31′′ S; 49° 08′ 51′′ W
HDB175	SAMN05194654	21.95	22° 54′ 02′′ S; 49° 10′ 23′′ W
HDB302	SAMN05194663	23.25	22° 52′ 02′′ S; 49° 09′ 07′′ W
HDB187	SAMN05194657	21.09	22° 53′ 56′′ S; 49° 10′ 24′′ W
HDB191	SAMN05194659	33.12	22° 54′ 02′′ S; 49° 10′ 23′′ W
HDB189	SAMN05194658	25.23	22° 54′ 02′′ S; 49° 10′ 23′′ W
HDB288	SAMN05194662	13.74	22° 50′ 31′′ S; 49° 08′ 51′′ W
HDB195	SAMN05194660	20.51	22° 52′ 02′′ S; 49° 09′ 07′′ W
HDB148	SAMN05194652	25.31	22° 54′ 02′′ S; 49° 10′ 23′′ W
HDB150	SAMN05194653	24.98	22° 53′ 56′′ S; 49° 10′ 24′′ W
HDB183	SAMN05194656	6.82	22° 50′ 31′′ S; 49° 08′ 51′′ W
HDB30-S	SAMN05194665	16.55	22° 52′ 02′′ S; 49° 09′ 07′′ W
LDB6	SAMN05194666	18.04	22° 54′ 02′′ S; 49° 10′ 23′′ W
LDB127	SAMN05194667	18.68	22° 53′ 56′′ S; 49° 10′ 24′′ W
LDB136	SAMN05194668	18.81	22° 50′ 31′′ S; 49° 08′ 51′′ W
LDB29	SAMN05194669	17.73	22° 54′ 02′′ S; 49° 10′ 23′′ W
LDB162	SAMN05194670	23.04	22° 54′ 02′′ S; 49° 10′ 23′′ W
LDB9	SAMN05194671	21.99	22° 54′ 02′′ S; 49° 10′ 23′′ W
LDB153	SAMN05194672	19.96	22° 53′ 56′′S; 49° 10′ 24′′ W
LDB8	SAMN05194673	15.48	22° 50′ 31′′ S; 49° 08′ 51′′ W
LDB23-S	SAMN05194674	30.04	22° 52′ 02′′ S; 49° 09′ 07′′ W
LDB181	SAMN05194675	25.04	22° 54′ 02′′ S; 49° 10′ 23′′ W
LDB35-S	SAMN05194676	18.42	22° 50′ 31′′ S; 49° 08′ 51′′ W
LDB40-S	SAMN05194677	21.15	22° 54′ 02′′ S; 49° 10′ 23′′ W
LDB196	SAMN05194678	17.18	22° 52′ 02′′ S; 49° 09′ 07′′ W
LDB5	SAMN05194679	12.11	22° 50′ 31′′ S; 49° 08′ 51′′ W
LDB221	SAMN05194680	13.64	22° 52′ 02′′ S; 49° 09′ 07′′ W
